# DeepLRHE: A Deep Convolutional Neural Network Framework to Evaluate the Risk of Lung Cancer Recurrence and Metastasis From Histopathology Images

**DOI:** 10.3389/fgene.2020.00768

**Published:** 2020-08-25

**Authors:** Zhijun Wu, Lin Wang, Churong Li, Yongcong Cai, Yuebin Liang, Xiaofei Mo, Qingqing Lu, Lixin Dong, Yonggang Liu

**Affiliations:** ^1^Department of Oncology, The First People’s Hospital of Changde City, Changde, China; ^2^Department of Oncology, Hainan General Hospital, Haikou, China; ^3^Sichuan Cancer Hospital and Institute, The Affiliated Cancer Hospital, School of Medicine, UESTC, Chengdu, China; ^4^Sichuan Cancer Hospital, Chengdu, China; ^5^Geneis (Beijing) Co., Ltd., Beijing, China; ^6^The First Hospital of Qinhuangdao, Qinhuangdao, China; ^7^Baotou Cancer Hospital, Baotou, China

**Keywords:** lung cancer, recurrence, hematoxylin and eosin staining, histopathological image, convolutional neural network

## Abstract

It is critical for patients who cannot undergo eradicable surgery to predict the risk of lung cancer recurrence and metastasis; therefore, the physicians can design the appropriate adjuvant therapy plan. However, traditional circulating tumor cell (CTC) detection or next-generation sequencing (NGS)-based methods are usually expensive and time-inefficient, which urge the need for more efficient computational models. In this study, we have established a convolutional neural network (CNN) framework called DeepLRHE to predict the recurrence risk of lung cancer by analyzing histopathological images of patients. The steps for using DeepLRHE include automatic tumor region identification, image normalization, biomarker identification, and sample classification. In practice, we used 110 lung cancer samples downloaded from The Cancer Genome Atlas (TCGA) database to train and validate our CNN model and 101 samples as independent test dataset. The area under the receiver operating characteristic (ROC) curve (AUC) for test dataset was 0.79, suggesting a relatively good prediction performance. Our study demonstrates that the features extracted from histopathological images could be well used to predict lung cancer recurrence after surgical resection and help classify patients who should receive additional adjuvant therapy.

## Introduction

Lung cancer accounts for 13% of newly diagnosed cancer incidences worldwide, resulting in 1.4 million deaths annually (Travis et al., 2011). According to the American Joint Committee on Cancer (AJCC), the TNM staging system is widely used for describing the anatomical extent of the disease on the basis of the assessment of three components: the extent of the primary tumor (T), presence and extent of regional lymph node metastasis (N), or presence of distant metastasis (M). The current TNM staging system is relatively accurate in defining the tumor stage. The recurrence rates of lung cancer patients in TNM stages I, II, and III are 34, 55, and 74%, respectively.

As is known to us all, the first-line treatment plan for a cancer patient is surgical removal of the primary tumor if there is no metastasis. However, the 5-year survival rate of postsurgical patients with early-stage lung cancer is only 54%, which is significantly worse than that of patients with breast cancer (∼90%) (Kaplan et al., 2016; Meng et al., 2018; Sun et al., 2019). One key factor leading to the poor postsurgical outcome for lung cancer patients is the loss of pulmonary function. Lobectomy leads to the loss or compromise of limited pulmonary function. On the other hand, wedge resections, which largely depend on the surgical resection margin, can save lung parenchyma but are associated with a nearly twofold increase in local cancer recurrence. It is crucial to decide the type of surgery to be performed because the 2-year survival rate in patients with local recurrence will drop to about 20% (Hung et al., 2009).

To alleviate the risk of surgical-related recurrence risk and increase the survival rate of postsurgical lung cancer patients, some invasive or non-invasive techniques have been used in clinical practice. First, the detection of circulating tumor cells (CTCs) at the time of surgery may represent an approach for identifying patients at a high risk of recurrence. A recent study indicated that the detection of pulmonary venous CTCs (PV-CTCs) at surgical resection could be used to evaluate future relapse (Chemi et al., 2019). Second, a few types of genomic alterations could be utilized to evaluate the risk of lung cancer recurrence owing to the strong association between genetic instability and tumorigenesis (Chan and Hughes, 2015). Next-generation sequencing (NGS) has a better testing performance with compatibility of low-input DNA. The National Comprehensive Cancer Network guideline of non-small-cell lung cancer recommended biomarkers favorable for target therapies such as epidermal growth factor receptor (EGFR) mutation (Couraud et al., 2014; Xu et al., 2016). Plasma and urine EGFR mutation levels could be used to predict the response of chemotherapy (Reckamp et al., 2016). NGS-based liquid biopsy is complemented with traditional tissue biopsy, which might be a promising strategy in the molecular profiling of lung cancer in the future. Furthermore, circulating tumor DNA and tissue assay might be combined to better predict lung cancer recurrence (Reckamp et al., 2016).

Since the rapid rise in the incidence and mortality of lung cancer, many researchers have shifted their focus on advanced discovery of novel diagnostic approach and predictive markers of metastasis, therefore, to assist clinical professionals to design individualized therapy for patients. Cancer recurrence following surgery or chemotherapy for lung cancer is a significant failure of local treatment as well as reduces the patient outcomes. Currently, cancer immunotherapy has been applied to cancer therapy. It has been recognized as adjuvant therapy for patients do not qualify for surgical intervention. The novel approach can be used to identify driver genes and predictive genes. For example, we have explained some lung cancer-specific gene mutation, gene sequencing, and biomarkers. PD-1 is an antibody against program death receptor and has been approved for second-line therapy of squamous cell carcinomas (Beer et al., 2002). Moreover, many preclinical trials demonstrated that combined traditional strategy and novel gene therapy may have improved patient overcome (Weiner et al., 2012).

Compared with other techniques, visual inspection of histologically stained slices is considered standard and used by pathologists to evaluate tumor stage, subtype, metastatic location, and prognosis (Fischer et al., 2008). With the absence of definitive pathological features, microscopic assessment requires experienced pathologists to evaluate stained slices. This process could be quite challenging and time-consuming for pathologists, and the results also depend on the quality of hematoxylin and eosin (H&E)-stained slices. Furthermore, accurate interpretation of an H&E image could be difficult because the distinction among different types of lung cancer is relatively unclear (MacConaill, 2013). To assist pathologists, deep learning tools have been developed to interpret the whole-slide image (WSI), which is helpful for developing an appropriate treatment plan and predicting survival outcomes. Yu et al. combined conventional image processing techniques with machine learning algorithms such as random forest, support vector machine, and naïve Bayes classifier to achieve acceptable prediction accuracy for lung cancer subtypes (Yu et al., 2016). The area under the receiver operating characteristic (ROC) curve (AUC) was approximately 0.75 in distinguishing two subtypes of lung cancer (Blumenthal et al., 2018). Furthermore, deep learning has also been successfully applied to the subtype classification of multiple cancers such as breast cancer, bladder cancer, and lung cancer (Zachara-Szzakowki et al., 2015; Araujo et al., 2017). The AUC reached approximately 0.83 by using The Cancer Genome Atlas (TCGA) dataset (Zachara-Szzakowki et al., 2015). Convolutional neural network (CNN) approach is not only used in cancer field, but it has been used in biochemical field as well. CNN has also served as a powerful approach to identify specific proteins located in electron transport chain, achieving good sensitivity (0.83%), specificity (94.4%), and accuracy (92.3%). This study demonstrated that the CNN approach can also be used in understanding the biochemical mechanism of important proteins such as electronic (Le et al., 2017, 2019). The same study team also used CNN to identify fertility related protein, which also received good sensitivity, specificity, and accuracy. Fertility-related proteins have critical function in reproductive organs and hormone-related fertility (Le, 2019). In fact, deep learning-based annotations of medical images are now close to, if not better than, those of pathologists for many types of cancers at present. With the development of image segmentation techniques (Simon et al., 2018), the WSI has been widely used for nuclei identification, tissue segmentation, and epithelial tissue identification in several cancers such as renal cancer, bladder cancer, and breast cancer (de Bel et al., 2018).

In this study, we established a novel machine learning framework to predict lung cancer recurrence by using the H&E-stained histopathological images. We first patched the H&E WSI into images of the size 512 × 512 pixels, which were then subject to a few image preprocessing steps such as image quality control and normalization. We then established a lung cancer tumor region prediction model and a cancer recurrence prediction model on the basis of the patched images. The prediction results based on patched images of a WSI were then combined to evaluate the recurrence risk of a lung cancer patient. Our model is cost-effective and could meet large clinical demands.

## Materials and Methods

### Data Preparation

**FIGURE 1:**
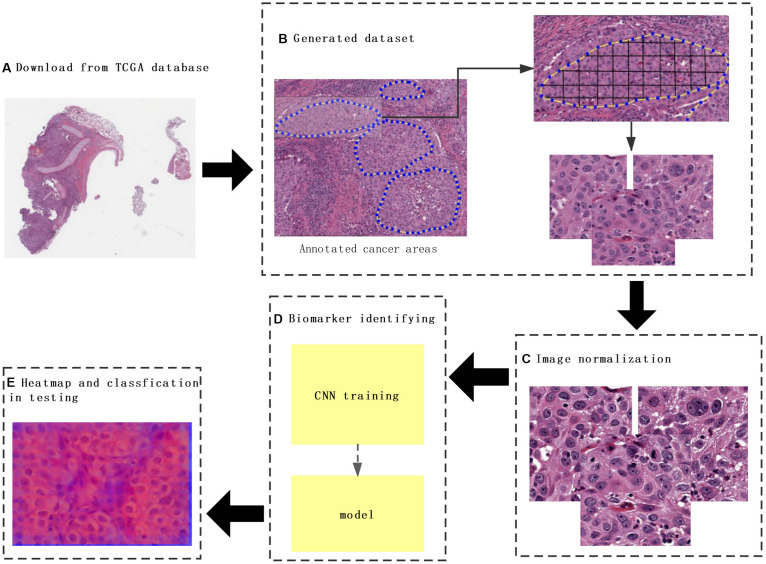
The flowchart of this study. **(A)** The whole-slide images (WSIs) of lung cancer downloaded from The Cancer Genome Atlas database. **(B)** Construction of a dataset consisting of annotated WSIs split by non-overlapping 512 × 512 pixels windows. **(C)** Color normalization. **(D)** Convolutional neural network (CNN) model training. **(E)** Heat map and classification of a testing sample. Each tile from the test image was classified by trained CNN, and the results were finally aggregated per slide to extract the heat map.

Hematoxylin and eosin images and clinical data of lung cancer were downloaded from TCGA database^1^, which is a landmark cancer genomics program that characterized thousands of primary cancers and matched normal samples spanning many cancer types. The labels that matched H&E images downloaded from TCGA contained information about metastasis and recurrence, and H&E image with SVS format was analyzed by the Python package OpenSlide. H&E images from those patients with the risk of metastasis and recurrence were labeled as “1” and “0” for those without metastasis and recurrence (Figure 1A).

### Image Preprocessing

**FIGURE 2:**
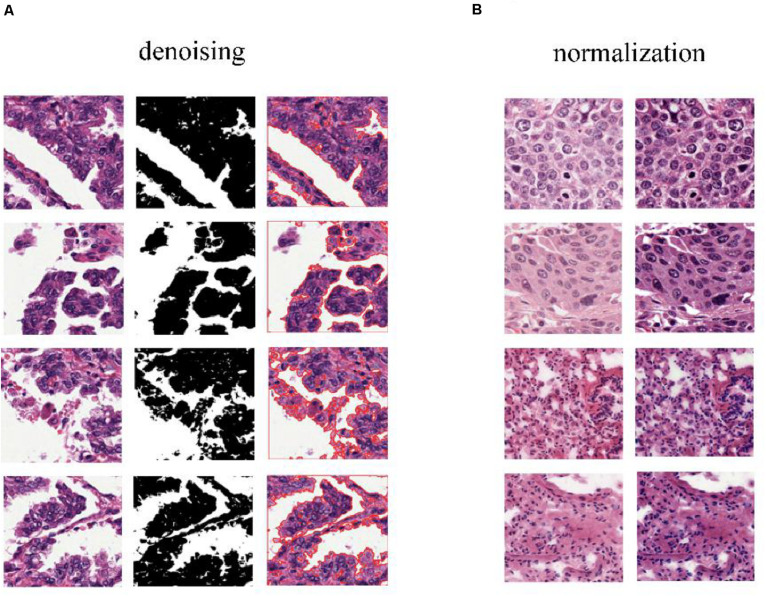
Color normalization of H&E slices. **(A)** The de-noising process applied to regions that have large blank spaces in the tumor regions. **(B)** The deep convolutional Gaussian mixture model (DCGMM) used for color normalization. The left column represents original images, and the right column represents imaging after color normalization.

To predict cancer recurrence and metastasis, tumor regions were annotated with the help of an expert pathologist by visual assessment. The morphology, color, and size of the nucleus of tumor cells are shown inside of tumor region, with the blue solid dotted lines representing the boundary of tumor (Figure 1B). For image preprocessing, each WSI was divided into computationally memory-affordable tiles of 512 × 512 pixels as input dataset. For noise reduction, Python’s OpenCV (version 4.1.1) package was applied to remove blank or blurred spaces in tumor region and to help reduce non-association interference in model training process. The non-association region was calculated as the ratio of the blank area or blurred spaces to the total area. The defined threshold of ratio was used to remove false-positive structures by definitive cutoff threshold. Further analysis of segmentation of H&E slice was performed by image de-noising, filtering, edge detection, expansion, and contraction techniques with OpenCV package (Figure 2A).

The performance of the computational technique for H&E-stained tissue image analysis is compromised by variable image colors due to H&E reagent concentration, staining process, and absorption caused by tissue fixation and staining method. To remove potential influenced variables, multiple color normalization (CN) approaches have been established (Vahadane et al., 2016), and unsupervised generative neural networks were applied in our study for performing stain-CN based on deep convolutional Gaussian mixture models (DCGMMs) in the stained H&E images (Shen et al., 2017; Wang et al., 2017; Qaiser et al., 2018; Simon et al., 2018). The DCGMM represents parameters of a fully CNN that are combined with the GMM parameters to optimize CN (Figure 2B).

### The Convolutional Neural Network + ResNet Model

In our study, Tensorflow 2.0.0 package was applied to conduct our model. To be specific, CNN was used effectively to identify tumor diagnoses by analyzing H&E-stained slices. CNNs are the most popular deep learning models for processing color images. The CNN deep learning network includes the input layer, intermediate hidden layer, and output layer. The intermediate hidden layer consists of multiple convolutional layers and pooling layers followed by more fully connected layers. The CNN could adapt and extract the feature hierarchy and classify images by error back propagation, which is a relatively effective gradient descent algorithm to update the weights connecting its inputs to the outputs during the training process.

After being transformed from the input layer, the image data were trained sequentially into the convolution layer composed of 32 *n* × *n* convolution kernels (e.g., *n* = 5) and the pooling layer for dimensional reduction through the ReLU excitation layer. The data were output to complete the entire feature extraction process afterward. Then, the data entered the second and third intermediate hidden layers, respectively. After the entire process was completed, all the features were extracted completely.

Batch normalization layer was then applied with the CNN to improve the generalization ability of the network and to expedite the training for higher learning rate. Increasing the number of layers of a deep CNN after reaching a certain depth could not improve the classification performance further, resulting in slower network convergence and worse classification accuracy due to the disappearance gradient problem.

**FIGURE 3:**
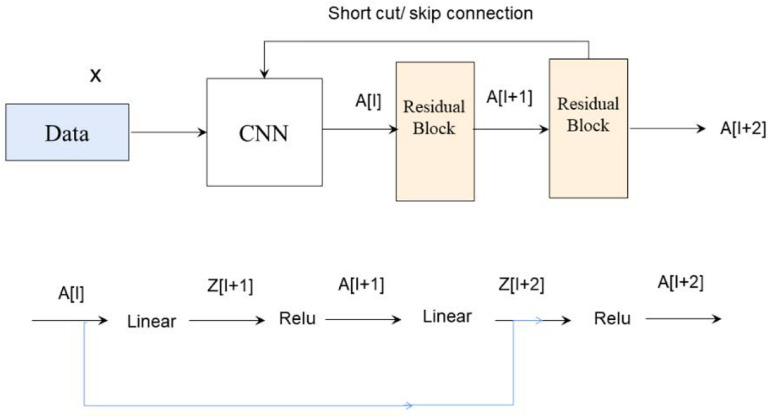
The ResNet network workflow.

ResNet was introduced to deal with this problem. The difference between residual and ordinary networks is the introduction of jump connection that can make the information of the previous residual block flow into the next one unimpeded, improve the information flow, and also avoid the disappearance gradient problem and the degradation caused by over depth of the network. Suppose there is a large neural network called big NN and its input is x and its output activation value is A[l]. After increasing the depth of the network, adding two additional layers to the network, and receiving the final output as A[l + 2], these two layers could be regarded as a residual block with a shortcut connection, and the activation function used in the whole network is relu. The function of relu is written by g(x). Linear function is written by W ^∗^ A + B. We can get A[l + 2] = g(Z[L + 2] + A[l]), where Z[L + 2] = W[l + 2] ^∗^ A[l + 1] + B[l + 1]. If W[l + 2] = 0, B[l + 1] = 0, we can know A[l + 2] = g(A[I]) then. When A[l] ≥ 0, A[l + 2] = A[l]. This is equivalent to establishing the linear relationship between A[l] and A[l + 2], when W and B is 0. It is equivalent to neglecting the two neural layers behind A[l] and realizing the linear transfer of the interlayer. The model itself can tolerate the deeper network, and this extra residual block will not affect its performance, and the relations are shown in Figure 3.

In fact, the residual network is composed of several shallow networks, and a shallow network could avoid the appearance of the vanishing gradient problem during training, thus accelerating the convergence of the network.

### Heat Map Generation

**FIGURE 4:**
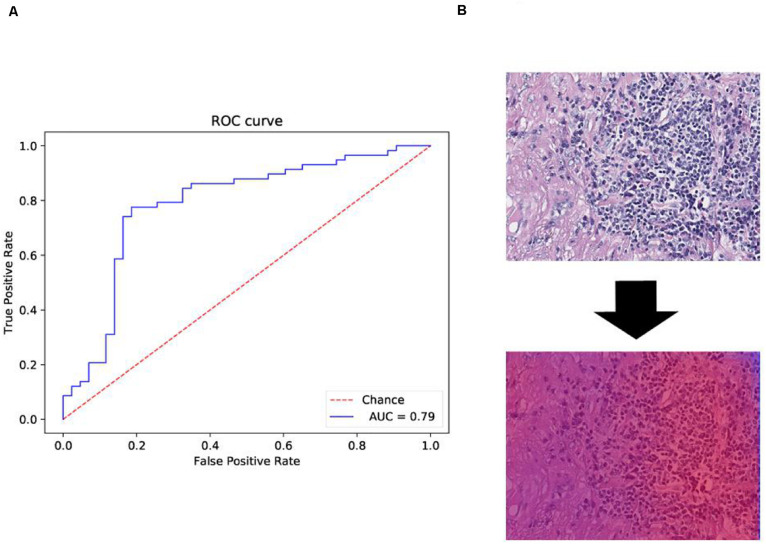
Receiver operating characteristic (ROC) and heat map on The Cancer Genome Atlas (TCGA) training data. **(A)** ROC curve of test data with the 512 × 512 pixel image. **(B)** Heat map of the tumor region applied in the convolutional neural network (CNN) model by using TCGA dataset. We also obtained the heat map given by the model shown in **B**. From the heat map, we found that the color of suspected tumor area was red and that the color of normal area was partial blue. The results were consistent as we have considered.

The probability maps were generated from the tumor region for high metastasis score detection (Figure 1E). The color in the probability map as shown in Figure 4B indicates the predicted metastasis score by pixels in the tumor region. The red color represents a high score, and blue color indicates a low score. H&E images were scanned by a 512 × 512 window in a step-wise manner, and results were obtained by the CNN model at each window. We applied the results on the pixels that were included in the window. We summed up all the values that pass the pixel and determined their average value, which was the predicted metastasis score of the pixel. The probability to recurrence and metastasis of every pixel was turned into color value with clear probability visualization. The probability value was mapped in the range of (0, 1) to RGB color from pure blue color (0, 0, 255) to pure red color (255, 0, 0) linearly. As a result, the red pixel image represents as a lower risk of metastasis; meanwhile, the light blue pixels represent no risk of metastasis, as shown in Figure 4B.

The WSI was divided into tiles, and each tile gets a probability result by model prediction during window sliding. Results of all tiles were integrated by fusion algorithm and computed as the final probability results for a specific slide. The average probability of top *n* windows was defined as identification score. Identification score predicted the risk of recurrence and metastasis with specific cutoff threshold. Scores higher than threshold were interpreted as positive results, whereas the top number value served as a hyper-parameter and is decided by cross-validation.

### Hyper-Parameter Tuning by Cross-Validation

A fivefold cross-validation was applied to prevent overfitting and to select hyper-parameters of the model for selecting the hyper-parameter space with best cross-validation score. The hyper-parameters that we tried to use in our model are activation function, patch, and top number. Our workflow is shown in the following three steps:

(1) Defining a grid on three dimensions with each of these maps for a hyper-parameter; for example, *n* = (activation function, patch, top number).
(2) For each dimension, defining the range of possible values.
(3) Searching for all the possible configurations and waiting for the results to establish the best one.

### Performance Evaluation Criteria

Several well-established performance evaluation criteria were employed to evaluate the performance of the classification model, including sensitivity (Se) or recall, specificity (Sp), precision, and the AUC.

$$\mathrm{Se}=\frac{\mathrm{TP}}{\mathrm{TP}+\mathrm{FN}}$$

$$\mathrm{Sp}=\frac{\mathrm{TN}}{\mathrm{TN}+\mathrm{FP}}$$

$$\mathrm{Precision}=\frac{\mathrm{TP}}{\mathrm{TP}+\mathrm{FP}}$$

$$\mathrm{F1}=\frac{2\times \mathrm{precision}\times \mathrm{recall}}{\mathrm{precision}+\mathrm{recall}}$$

In the equations, TP stands for the number of images correctly recognized as positive samples. FP stands for the number of images that were incorrectly recognized as positive samples. FN stands for the number of images incorrectly recognized as negative samples. TN stands for the number of images correctly recognized as negative samples. We indicate TP, FP, TN, and TP by confusion matrix as shown in Table 1.

**TABLE 1:** Confusion matrix definitions.

**Confusion matrix**		**Prediction**	
		**Positive**	**Negative**
True	Positive	True positive (TP)	False negative (FN)
	Negative	False positive (FP)	True negative (TN)

## Results

### Clinical Characteristics of Training Dataset

A total of 110 H&E images of lung cancer patients with metastasis or recurrence information were downloaded from TCGA, and the available datasets were selected with required condition with data type of slide image, data format of SVS, primary site for bronchus and lung, and white ethnicity (Table 2). The average age of the selected patient cohort was 54 years, and 68% of the patients have metastasis or recurrence. We labeled data to positive with new_tumor_event_type of Distant Metastasis and Locoregional Recurrence. At the same time, we labeled data to negative with tumor_status of Tumor Free.

**TABLE 2:** Clinical characteristics.

**Clinical variable**	**Category**	**Clinical level**
Age	Mean	54 (31–83)
Gender	Male	62
	Female	47
	Unknown	1
Samples type	H&E	1
Metastasis and recurrence period	Tumor Free	35
	Loco regional recurrence	15
	Distant metastasis	60
Cancer subtype	Adenocarcinoma	58
	Squamous carcinoma	52

### Data Pre-treatment

The 110 H&E images with corresponding clinical data downloaded from TCGA are all in SVS format. Whole images could not be used as the input data for the network. Hence, we segmented them into tiles with a 512 × 512 pixel size from the 110 H&E images in which tumor regions were annotated in the WSIs by the expert pathologist. The tiles with a low amount of information (e.g., more than 70% of the surface was covered by background) were removed. Thereafter, a template image was selected by an expert pathologist. Then we trained the DCGMM by using this template image. After training, we applied the model on the H&E image on the upper row of the compared color normalized image down the row (Figure 1C). The results are shown in Figure 2B.

### Model Construction and Hyper-Parameter Selection

DeepLRHE model was constructed with ResNet network and top five selection algorithm of WSI (Figure 1D). We used GridSearchCV class in scikit-learn by providing a dictionary of hyper-parameters to determine the hyper-parameters of the model. The hyper-parameters we selected are shown as follows:

Top⁢number=[5,3]

Patch=[100,150,200]

Activation⁢function=[softmax,relu,tanh].

After the cross-validation process, activation function is set to relu, patch number is set to 150, and top number is set to 5 as shown in Table 3, in which performance is the best (the AUC value reached a maximum value of 0.84). We used selected hyper-parameters to construct the DeepLRHE model on whole 110 training dataset.

**TABLE 3:** Tuning of the hyper-parameters.

**Activation function**	**Patch**	**Top number**	
		**5**	**3**
softmax	100	0.79	0.73
	150	0.82	0.75
	200	0.82	0.78
relu	100	0.81	0.74
	150	0.84	0.78
	200	0.83	0.80
tanh	100	0.73	0.6
	150	0.76	0.67
	200	0.77	0.69

### Performance Evaluation on Test Dataset

Another 101 H&E images were downloaded with their clinical information in different project from train dataset in TCGA. The datasets were available on TCGA in condition with data type of slide image, data format is SVS, and primary site of bronchus and lung and ethnicity is not reported (this condition is different from that of the training dataset). The trained model was applied on those data and obtained the confusion matrix below and ROC curve in Figure 4A.

The performance evaluation results were calculated from the confusion matrix in Table 4. The results showed that the sensitivity and specificity of the model were 0.84 and 0.67, respectively. The precision and F1 score reached 0.78 and 0.81, respectively, in the independent test dataset. In the meantime, the model achieved 0.79 AUC score. The AUC value on independent test dataset was lower compared with AUC value (0.79 vs. 0.84) of fivefold cross-validation method on train dataset. The performance evaluation from independent test dataset was more convincing.

**TABLE 4:** The confusion matrix of the model for test dataset.

**True**	**Prediction**		**Total**
	**High risk**	**Low risk**	
High risk	49	9	58
Low risk	14	29	43
Total	63	38	101

## Discussion

Machine learning algorithms have been widely used in clinical practice. They can map unstructured information into a structured form as well as enable automatic identification and extraction of relevant information. Such an automated system enables us to significantly reduce time-consuming diagnostic procedures. With a dramatic improvement in the affordability of the testing, it has also brought challenges pertaining to the evaluation of effectiveness and accuracy of gene testing, which could affect diagnosis and subsequent therapy. Therefore, machine learning algorithms have been a hot topic and a dynamically changing area in the recent years. Therefore, these models require human experts to encode the domain knowledge through feature engineering. However, the results of such models are still controversial and time dependent.

Recently, multilayer NNs or deep learning has been applied to gain insights from heterogeneous clinical data. The major difference between deep learning and conventional NN is the number of hidden layers as well as their capability to learn meaningful abstractions of the input. Deep learning has been applied to process aggregated clinical documents (imaging, pathological slices from biopsy, and other reports). Several studies have used deep learning to predict disease prognosis from medical documentation; for example, one study used a four-layer CNN to predict congestive heart failure and chronic obstructive pulmonary disease that showed promising performance. CNN is a powerful algorithm for advancing biomedical images and analysis (Makowski and Hayes, 2008; Shackelford et al., 2013). It can be applied for pathological image analysis tasks such as tumor detection and quantification of cellular features by using either general staining slices or in combination with immunohistological markers (Mogi and Kuwano, 2011; Morris et al., 2013). Computerized image processing histopathological analysis system has been impressive in the prognostic determination of various tumors and even precancerous lesions in the esophagus (Chiang et al., 2016). Recent studies showed that many histological features are associated with survival outcomes. Deep learning tumor detection allows for tumor size calculation and shape estimation. Tumor size and shape are a well-established prognostic marker for lung cancer, and the boundary of the tumor region has been reported to be associated with a poor local prognosis marker as well (Esteva et al., 2017). Furthermore, most tumor-related features including the tumor area, perimeter, convex area, and filled area of the tumor region were associated with poor survival outcome (Popin et al., 2018). Extracting tumor features from H&E were usually conducted by experienced experts; however, the extraction process is subject to human bias and is time-consuming. CNNs, as the most popular deep learning model for imaging processing, could directly handle multidimensional color image and extract the regional boundary of the pixels. Moreover, CNNs can retain parameters during imaging processing as well as effectively identify similar images.

In this study, to identify tumor regions, the pathological images were divided into 512 × 512 pixel patches to classify as tumor, non-malignant, or white categories using the CNN model. The CNN model was trained on image patches that were downloaded from TCGA database for lung squamous cell carcinoma (LUSC). Moreover, we compared the performance of our model on the test set with the performance of experienced pathologists. Our results reached an 81% AUC score. Moreover, our model has strong generalizability for learning comprehensive tissue and cell morphological changes that could be used as an auxiliary approach to make a pathological diagnosis for different types of cancers. Also, our results suggest that deep learning of histopathological imaging features can predict the prognosis of lung cancer patients, thereby assisting health professionals to make precision treatment plans.

Our study has several limitations. TCGA images exclusively composed of lung adenocarcinoma (LUAD) cells, LUSC cells, or normal lung tissues. However, several images contain features that the model has not been trained to recognize, making the classification task more challenging. For example, we observed several non-specific features including blood vessels, inframammary cell infiltration, and necrotic regions in the lung tissue as well as bronchial cartilage and fibrous scars. Moreover, this study did not include an independent set to validate our model, which may have compromised the accuracy of the results.

Overall, here, we established a novel deification model for pathological diagnoses. This model interpreted predictions through convolutional natural language and visual attention that could help pathologists to analyze histological slices. Our model could allow diagnostic consistency and establish cost-effective systems to meet large clinical demands with less manual intervention and time efficiency by analyzing precise pixels objectively. Future studies are necessary to testify its performance for other types of cancers such as gastrointestinal cancers.

## Data Availability Statement

All datasets presented in this study are included in the article/supplementary material.

## Author Contributions

YBL and LD designed the project. ZW, LW, CL, YC, YBL, XM, and QL analyzed the data, performed the experiments, and wrote the manuscript. ZW and YGL modified and reviewed the manuscript. All authors contributed to the article and approved the submitted version.

## Conflict of Interest

YBL, XM, and QL are employed by the company Geneis (Beijing) Co., Ltd.

The remaining authors declare that the research was conducted in the absence of any commercial or financial relationships that could be construed as a potential conflict of interest.

## Footnotes

1 https://portal.gdc.cancer.gov/repository/
